# Frequency comb-to-comb stabilization over a 1.3-km free-space atmospheric optical link

**DOI:** 10.1038/s41377-022-00940-3

**Published:** 2022-08-12

**Authors:** Jaewon Yang, Dong IL Lee, Dong-Chel Shin, Jaehyun Lee, Byung Soo Kim, Hyun Jay Kang, Young-Jin Kim, Seung-Woo Kim

**Affiliations:** 1 Department of Mechanical Engineering, Korea Advanced Institute of Science and Technology (KAIST), 291 Daehak-ro, Yuseong-gu Daejeon, 34141 Republic of Korea; 2 Presently with Korea Research Institute of Standards and Science (KRISS), 267 Gajeong-ro, Yuseong-gu Daejeon, 34113 Republic of Korea

**Keywords:** Frequency combs, Optical metrology

## Abstract

Stabilizing a frequency comb to an ultra-stable optical frequency reference requires a multitude of optoelectronic peripherals that have to operate under strict ambient control. Meanwhile, the frequency comb-to-comb stabilization aims to synchronize a slave comb to a well-established master comb with a substantial saving in required equipment and efforts. Here, we report an utmost case of frequency comb-to-comb stabilization made through a 1.3 km free-space optical (FSO) link by coherent transfer of two separate comb lines along with a feedback suppression control of atmospheric phase noise. The FSO link offers a transfer stability of 1.7 × 10^–15^ at 0.1 s averaging, while transporting the master comb’s stability of 1.2 × 10^–15^ at 1.0 s over the entire spectrum of the slave comb. Our remote comb-to-comb stabilization is intended to expedite diverse long-distance ground-to-ground or ground-to-satellite applications; as demonstrated here for broadband molecular spectroscopy over a 6 THz bandwidth as well as ultra-stable microwaves generation with phase noise of -80 dBc Hz^–1^ at 1 Hz.

## Abstract (web summary)

Our remote comb-to-comb stabilization expedites diverse long-distance ground-to-ground or ground-to-satellite applications for 6 THz bandwidth molecule spectroscopy and microwaves generation with phase noise of −80 dBc Hz^–1^ at 1 Hz.

## Graphical abstract

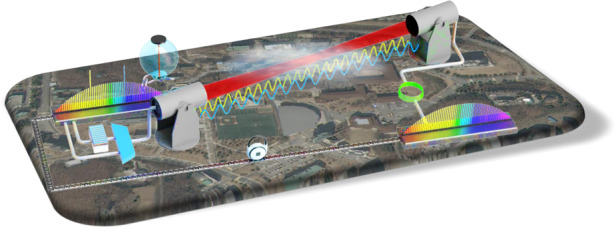

## Introduction

The optical spectrum of a mode-locked ultrashort pulse laser, routinely called the frequency comb for its unique structure comprised of many evenly-spaced discrete spectral lines, has been enabling breakthroughs in diverse fields of precision metrology over the past few decades ^1–4^. Such remarkable advancement began with stabilizing the frequency comb with reference to the radio-frequency (RF) atomic clock through two collective parameters; the pulse repetition rate and the carrier-envelope offset frequency ^5^. Afterward, with the advent of optical clocks, the comb stabilization has evolved to the optical regime with improved frequency stabilities to a few mHz level or below^6–8^. The practice of comb stabilization to an optical clock, however, requires complicated optoelectronic peripherals, such as narrow-linewidth probing lasers and an auxiliary optical cavity of short-term stability, which even have to be operated altogether under strict ambient control^9–11^. The increased hardware burden consequently restricts the comb stabilization to be implemented within a well-equipped laboratory condition, thereby the comb line frequencies are disseminated outside through an optical fiber network with appropriate phase noise suppression^12,13^.

The method of fiber-network-based optical frequency transfer over a long distance has been well established as verified in the transmission of high-accuracy optical clock signals for remote clock-to-clock comparisons^14,15^ and relativity experiments^7,8^. Meanwhile, the ability to transfer optical frequencies via a free-space optical (FSO) link has also been indispensable especially for inter-continental scale, ground-to-satellite, and space-borne applications^8,16–20^. If the optical clock signal is to be delivered without the loss of its original stability through open air, the FSO link needs to be well equipped with the functional capabilities of pointing, acquisition, and tracking (PAT)^21,22^, but also active suppression of the atmospheric phase noise caused by ambient temperature and pressure fluctuation^23^. In the framework, an 18 km FSO link was recently demonstrated by the authors to transfer optical frequencies with a 10^–15^ fractional instability at 0.1 s averaging^24^. The demonstration was intended particularly to utilize the frequency comb as a versatile light source to generate optical carrier signals and at the same time as a frequency ruler to compensate for the atmospheric phase noise^24,25^.

In this Letter, we describe an extended application of the FSO link made for the optical frequency comb-to-comb stabilization to synchronize a slave comb with a master comb over a 1.3 km atmospheric open path. The master comb is put in stabilization to a high-finesse optical resonance cavity made of very low thermal expansion material to offer a supreme short-term optical frequency stability. The FSO link is established by coherent transmission of two separate comb lines extracted from the master comb directly by means of injection locking. During transmission through the open air, the atmospheric phase noise is suppressed by real-time feedback control. This comb-to-comb stabilization through the FSO link is intended to expedite the atmospheric transfer of optical clock signals for long-distance ground-to-ground or even ground-to-satellite applications. As exemplary studies, the remotely stabilized slave comb is employed for high-precision molecular spectroscopy, 10 GHz microwaves generation, and 0.1 THz millimetre waves detection.

## Results

### FSO link configuration

**Fig. 1:**
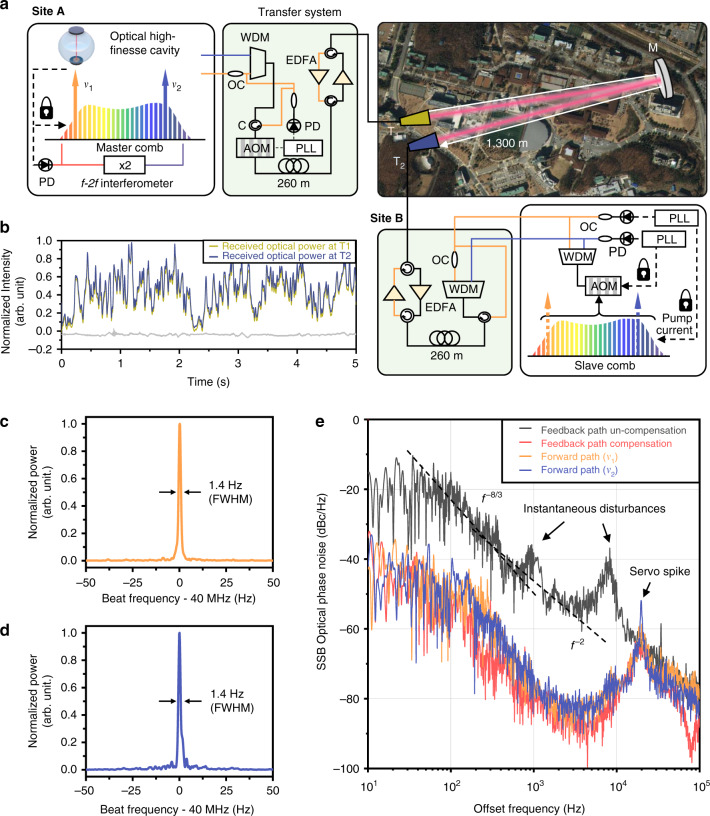
Frequency comb-to-comb stabilization over a 1.3 km free-space optical link. **a** Overall system configuration. The master comb is located at Site A in stabilization to a high-finesse optical cavity. A slave comb to be stabilized is placed at Site B. Two optical frequencies ν_1_ (1531 nm) and ν_2_ (1564 nm) are extracted from the master comb at Site A, transferred to Site B through a free-space optical link. **b** Time-dependent power fluctuation of the optical carrier signals received by the transceivers; T_1_ at Site A and T_2_ at Site B. Grey line at bottom denotes the calculated power difference between T_1_ and T_2_. **c**, **d** Linewidths of the transferred optical frequencies ν_1_ and ν_2_, respectively, at Site B with phase noise suppression control. **e** Single sideband (SSB) phase noise spectra of forward path (one-way) and feedback (two-way) optical carrier signals. WDM Wavelength division multiplexer, AOM Acousto-optic modulator, C Circulator, EDFA Erbium-doped fiber amplifier, OC Optical coupler, PD Photodetector, T Transceiver, M Flat mirror, and PLL Phase-locked loop

Figure 1 shows the comb-to-comb stabilization system configured in this investigation over a 1.3 km atmospheric FSO link between Site A and Site B. The FSO link was folded in half by placing a flat mirror of a 120 mm aperture diameter in the middle, so Site B was kept close to Site A for a direct stability comparison between the two sites. The master comb was made of an Er-doped fiber oscillator (C-fiber, Menlosystems GmbH) at Site A with an elaborate scheme of stabilization to a high-finesse cavity, offering an overall comb stability of 3.8 × 10^–15^ at 0.1 s (Methods). The slave comb was set at Site B, which in principle can be an arbitrary type of frequency comb different from the master comb in terms of the repetition rate and spectral range. For stabilization of the slave comb, two separate comb lines, ν_1_ (1531 nm) and ν_2_ (1564 nm), were extracted from the master comb and sent to Site B over the FSO link. Specifically, each comb line was filtered out individually through a fiber Fabry-Perot etalon coupled with a fiber Bragg grating, and injection-locked to a distributed-feedback laser diode for power amplification to 20 mW without loss of the original frequency stability^25^.

The two extracted comb lines, ν_1_ and ν_2_, were combined into a single beam through a single-mode fiber, delivered via a fiber network of a 260 m length, and launched to Site B in the open air through a refractive-optic transceiver offering a 40 mm aperture diameter. Another identical refractive-optic transceiver was installed at Site B to receive the carrier beam by focusing it into a single-mode fiber. Power boosting was provided using a pair of Er-doped fiber amplifiers (EDFAs), one before launching at Site A and the other after receiving at Site B, to counterbalance the power attenuation encountered in the transceiver-to-transceiver open-air transmission plus the ground fiber networks of RF & optical signal processing (Methods).

In order to compensate for the phase noise confronted in atmospheric transmission, one comb line, ν_1,_ was partly sent back to Site A via the same FSO link along the reverse direction (Fig. 1a). The returned comb line was mixed with its original comb line so that the atmospheric phase noise was quantified in terms of the Doppler RF beat. Since it was well verified that the wavelength-dependent variation of the air refractive index is not significant in the 1550 nm near-infrared light band^24,26^, the comb line, ν_2_ was assumed to experience the same amount of Doppler shift as the comb line, ν_1_. Thus, both the comb lines were treated together, modulated using an acousto-optic modulator (AOM) to pre-compensate the Doppler RF beat of the comb line, ν_1_ by phase-locked loop (PLL) control. At the same time, at Site B, the slave comb was put on to stabilization by adjusting its overall comb spectrum to the transferred ν_1_ and ν_2_. Specifically, the two lowest interference RF beats between the slave comb and the transferred comb lines, ν_1_ and ν_2_ were filtered out simultaneously, locked to the pre-assigned values individually by activating two separate PLL controls^27^. One PLL control was performed by operating an acousto-optic modulator (AOM) and the other PLL control by regulating the pumping current to the fiber oscillator of the slave comb. The AOM control permits adjusting the lateral position of the whole comb structure in the spectral domain, thereby settling the carrier-envelope offset *f*_0_ of the slave comb to the desired value. The pump current control allows fine-tuning of the inter-mode spacing of the comb structure by regulating the repetition rate *f*_r_. In consequence, the dual-PLL control arrangement leads both *f*_0_ and *f*_r_ to converge to their desired values individually, which are not necessarily identical to those of the master comb.

### Reversal symmetry

For precise feedback compensation of the atmospheric phase noise, a prerequisite is that the FSO link has to be designed to maintain a high level of reversal symmetry between the forward path (from Site A to Site B) and the feedback path (from Site B to Site A). This reciprocity condition demands that the time-dependent fluctuation of the optical power finally collected at the transceiver show no difference between the forward path and the backward path, being affected only by instantaneous atmospheric turbulence alike. Hence, for our FSO link system, special care was given to the transceiver systems as well as the EDFA amplifiers at both the sites to be as much symmetrical as possible (Fig. 1a). As a result, the actually measured path-dependent disparity between the forward and backward paths (Fig. 1b) turned out to be as small as 0.9% in terms of the standard deviation of the received power fluctuation. Accordingly, the 1.4 Hz linewidth of the comb line ν_1_ monitored upon arriving at Site B via the forward path (Fig. 1c) was found to undergo no notable degradation when returned back at Site A via the feedback path, maintaining a 15-digit comb line stability (Fig. 1d).

For more elaborate analysis, the power spectral density (PSD) of the atmospheric phase noise was calculated by Fourier-transform of the Doppler RF beat of the returned comb line ν_1_ traced with respect to its original comb line (Fig. 1e, black line). The result revealed that when the feedback compensation control was not activated, the PSD was dominated by air turbulence particularly for low frequencies less than 1 kHz with a –8/3^th^ power slope as predicted by the theoretical Kolmogorov’s spectrum of weak turbulence^28^. For higher offset frequencies, a substantial amount of fiber-induced phase noise gradually emerged with a 2^nd^ power slope, together with the sudden appearance of small peaks attributable to localized air turbulence. Nonetheless, when the feedback compensation was activated with a control bandwidth of 20 kHz, the aforesaid atmospheric, as well as the fiber-induced phase noise, was suppressed (Fig. 1e, red line), for instance, to –40 dBc Hz^–1^ at 10 Hz and –80 dBc Hz^–1^ at 5 kHz. Note that our FSO link consists of a 1.3 km open-air path and an additional sum of 520 m fiber network line, which leads to a total round-trip time delay of 13.6 μs along with the appearance of a servo spike at a corresponding frequency of 37 kHz due to the intrinsic instability of delayed feedback control^29^. Indeed, the measured PSD plot (Fig. 1e, red line) showed an actual servo spike at a slightly lower frequency of 20 kHz, being reckoned attributable to some unaccounted time delays residing within the RF components used in our control electronics. Even in the presence of the servo spike, its influence on the final phase noise compensation was found to be minimal; the PSD plots of the comb lines of ν_1_ and ν_2_ (Fig. 1e, yellow & blue) monitored at Site B, after one-way travel of the forward path, yielded no notable difference from that of the compensated comb line, ν_1_ (Fig. 1e, red line). Note that the PSDs of ν_1_ and ν_2_ are converted by calculation to their corresponding residual instabilities of 1.5 × 10^–15^ and 1.7 × 10^–15^ at 0.1 s averaging, respectively.

### Evaluation of comb-to-comb stabilization

**Fig. 2:**
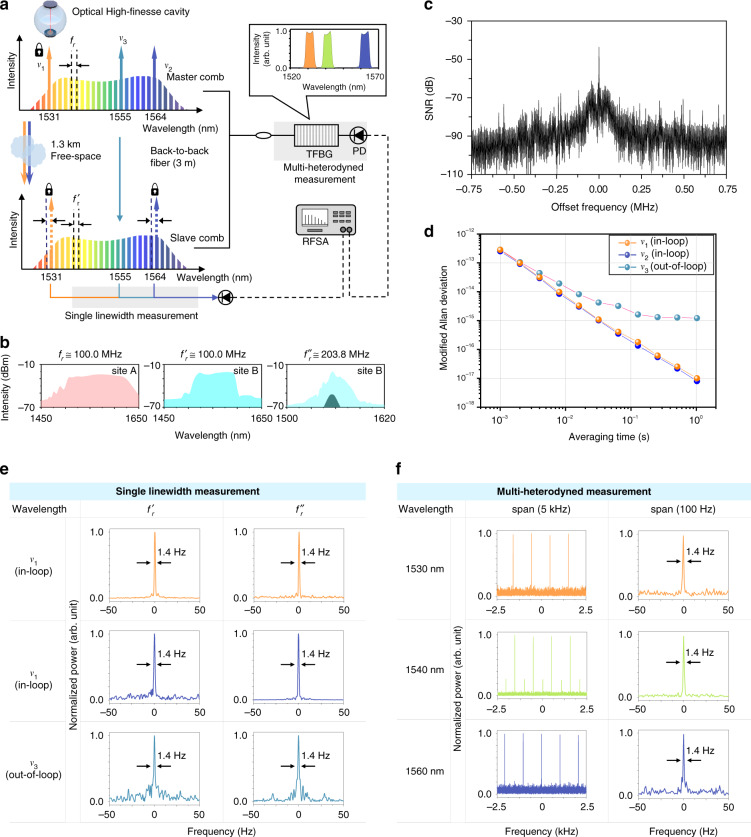
Mode-to-mode verification of comb-to-comb stabilization. **a** The slave comb is connected to the master comb through a short fiber line for back-to-back evaluation. Mode-to-mode relative stability is monitored for two in-loop frequencies (ν_1_, ν_2_) and one out-of-loop frequency (ν_3_). **b** Optical spectra of the master comb (*f*_*r*_ = 100.0 MHz) and two distinct slave combs (*f*_*r*′_ = 100.0 MHz and *f*_*r*″_ = 203.8 MHz). The original spectrum of *f*_*r*″_ is given in dark green while its broadened spectrum in light blue. **c** RF spectrum of the mode-to-mode error signal for the in-loop frequency ν_1_. **d** Allan deviations of the in-loop and out-of-loop frequencies. **e** Modal linewidth measurement. **f** Multi-heterodyne modal linewidth measurements at three different filter frequencies (orange:1530 nm, green: 1540 nm, blue: 1560 nm). TFBG Tunable fiber Bragg grating, PD Photo-detector, and RFSA Radio frequency spectrum analyzer

Figure 2a illustrates how the performance of the comb-to-comb stabilization implemented in this investigation was evaluated. As Site A and Site B were kept close by folding the FSO link in half, a direct comb line-to-line comparison was made between the master comb and the slave comb through a fiber line of a 3 m length. The master comb was set to have a repetition rate (*f*_*r*_) of 100.0 MHz. As for the slave comb, in order to demonstrate the operational flexibility of the comb-to-comb stabilization scheme proposed in this study, two distinct oscillators were tested; one was configured to operate at a repetition rate of *f*_*r*_′ = 100.0 MHz and the other at *f*_*r*_″ = 203.8 MHz. Note that comb-to-comb synchronization with different repetition rates was achieved by varying the RF beat offsets in the process of PLL locking of the slave comb to the transferred comb lines of ν_1_ and ν_2_ (Methods).

Now, having activated the FSO link along with the real-time feedback compensation of atmospheric phase noise (Fig. 1), the PLL locking stability of the slave comb with respect to the transferred comb lines was assessed by monitoring the in-loop error signals. The RF spectrum of the PLL error signal for ν_1_ showed a sharp coherent peak of a 37 dB signal-to-noise ratio (Fig. 2c), which was almost the case for ν_2_ as well without notable disparity. The residual PLL locking error was measured to be 1.0 × 10^–17^ and 8.0 × 10^–18^ at 1.0 s averaging for ν_1_ and ν_2_, respectively, in terms of the Allan deviation (Fig. 2d). This in-loop PLL data provides an overall performance index of the frequency transfer stability of the whole FSO link system. Secondly, a direct comb line-to-line comparative evaluation between the slave comb and the master comb was made; specifically, an arbitrary comb line of ν_3_ was extracted from the master comb and mixed with the slave comb. The subsequent lowest RF beat signal, representing a line-to-line comparison at an out-of-loop frequency of ν_3_, was traced to determine the relative stability of the slave comb with respect to the master comb. When the out-of-loop comb line ν_3_ was chosen at 1555 nm, in between the in-loop comb lines of ν_1_ (1531 nm) and ν_2_ (1564 nm), the relative stability was measured to be 1.2 × 10^–15^ at 1.0 s averaging in terms of the Allan deviation. This out-of-loop stability was two orders of magnitude larger than the residual PLL locking error of the in-loop comb lines. Nonetheless, it is important to note that the out-of-loop stability was on the same order as the original frequency stability of the master comb confirmed with reference to a high-finesse resonance cavity (Methods). This infers that the atmospheric phase noise was well suppressed in our remote comb-to-comb stabilization, causing no notable stability degradation on the slave comb in comparison with the master comb. Further, the line-to-line comparison revealed that the relative linewidths of the in-loop (ν_1_ and ν_2_) and out-of-loop (ν_3_) comb lines are evaluated equally to be 1.4 Hz (Fig. 2e). As a result, with the assumption of equal contribution of the two combs to the line-to-line linewidth, the slave comb as well as the master comb was estimated to have an individual linewidth of 1.0 Hz.

Lastly, for a more inclusive evaluation, multi-line interference was taken to verify the overall coherence of the slave comb with respect to the master comb. Specifically, a slight difference of 1.037 kHz was given to the 100.0 MHz repetition rate of the slave comb, while the master comb was set at a repetition rate of 100.0 MHz (Methods). For quantitative assessment, three distinct wavelength regions were selected at 1530 nm, 1540 nm, and 1560 nm through a tunable fiber Bragg grating (TFBG) filter with a spectral bandwidth of 5 nm per each. This dual-comb multi-heterodyne interference with subsequent RF spectrum analysis led to linewidth measurements over each wavelength region (Fig. 2f), indicating a common relative linewidth of 1.4 Hz alike without notable difference over the three-wavelength regions. This result confirmed that the slave comb was evenly well stabilized through the FSO link over a 4.2 THz spectral bandwidth.

### Calibration of molecular absorption spectra

**Fig. 3:**
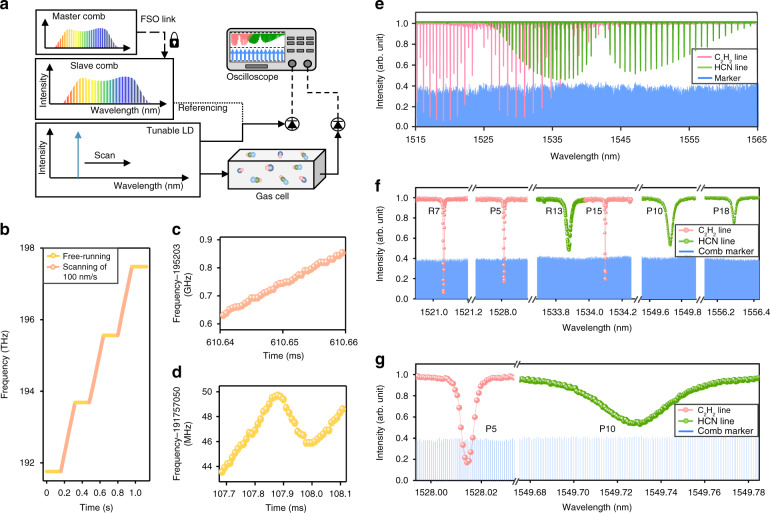
Molecular absorption spectroscopy using the slave comb. **a** System configuration to calibrate a tunable laser diode (LD) scanning molecular absorption spectra. **b** Optical frequency scanning from 191.7 THz to 197.7 THz with a tuning speed of 100 nm s^–1^. **c** Scanning state with frequency calibration. **d** Free-running state. **e** Molecular absorption spectra from ^12^C_2_H_2_ and H^13^C^14^N gas cells with calibration to the slave comb. **f** R7, P5, and P15 lines of ^12^C_2_H_2_ and R13, P10, and P18 lines of H^13^C^14^N gas cells. Absorption peaks and comb-referenced markers extracted in steps of 100 MHz. **g** Expanded views of P5 line of ^12^C_2_H_2_, P10 line of H^13^C^14^N, and comb markers

The usefulness of the remote comb-to-comb stabilization proposed in this study was corroborated by implementing molecular absorption spectroscopy using the slave comb^30,31^. Figure 3a illustrates the overall scheme of spectroscopy configured at Site B for the purpose, in which the slave comb acted as a frequency ruler. Specifically, a continuous-wave wavelength-tunable diode laser (Phoenix 1400, LUNA) was employed to produce the probe beam at a fast scanning speed of 100 nm s^–1^ over a wavelength range from 191.7 THz (1563.9 nm) to 197.7 THz (1516.4 nm) in three sequences (Fig. 3b–d). Prior to the wavelength scanning, the initial frequency of the probe beam was once identified using a wavelength meter (86122a, Agilent) certified with an absolute uncertainty of 37 MHz (0.3 pm). Then, for fast and precise measurement, the instantaneous frequency of the probe beam was calibrated during scanning by monitoring its beat frequency *f*_beat_ with the slave comb through a digital oscilloscope. The traced RF beat, *f*_beat_ underwent a repeated variation following a saw-tooth pattern with a periodicity equal to the repetition rate of the slave comb set at 100.0 MHz. Whenever the beat, *f*_beat_ crossed a pre-set threshold of near 30 MHz, electrical marker lines were created and stored within the digital oscilloscope, with which the measured absorption spectrum was rectified by post-processing calibration.

For actual evaluation, spectroscopic data were taken on two distinct molecular samples; acetylene (^12^C_2_H_2_) cell (2.67 kPa, 120 mm) and hydrogen cyanide (H^13^C^14^N) cell (13.3 kPa, 130 mm). The gas cells were installed back to back for simultaneous sampling, and their absorption spectra were recorded over the entire scanning range along with slave comb-referenced marker lines positioned uniformly with a spacing of 100 MHz (Fig. 3e). Then all absorption peaks were Voigt-fitted to locate their center wavelengths with a spectroscopic resolution of 0.001 pm with respect to the marker lines (Fig. 3f). The spectroscopic accuracy was verified representatively through the P5 line of ^12^C_2_H_2_ and the P10 line of H^13^C^14^N, of which the center wavelengths were determined to be 1528.014366 nm and 1549.720206 nm, respectively (Fig. 3g). The measured center positions were found to yield a small deviation of 0.041 pm and 0.062 pm, respectively, in comparison to the certified literature values provided in refs. ^32,33^. This confirmed that the remotely stabilized slave comb was able to act as a wavelength calibrator providing excellence in terms of the narrow linewidth, absolute uncertainty, calibration speed, and broadband wavelength coverage.

### Comb-based microwave generation

**Fig. 4:**
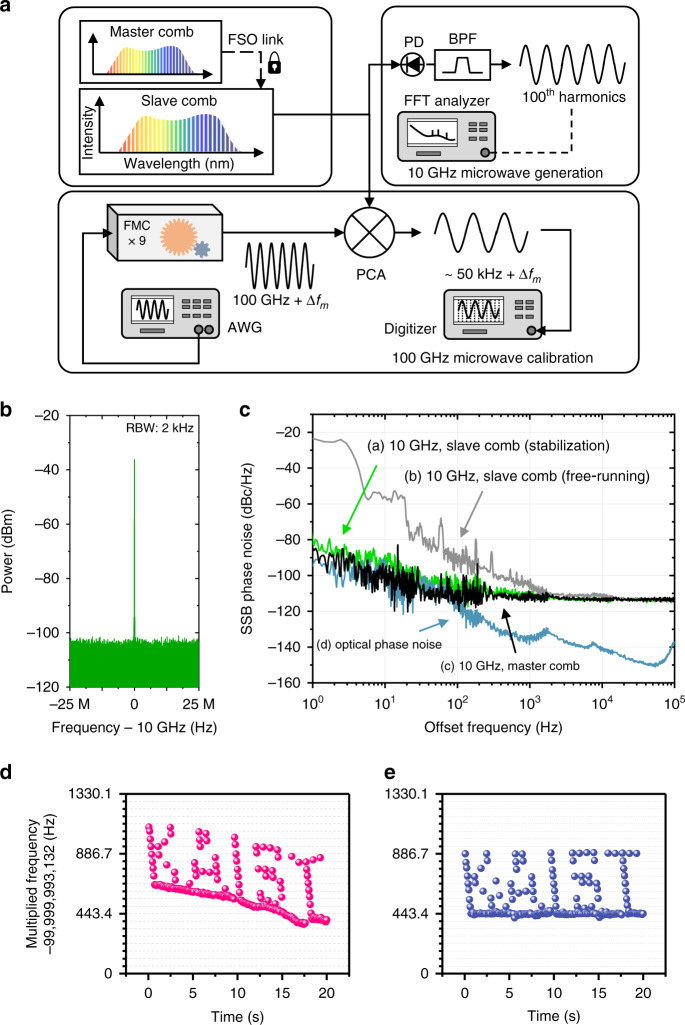
Comb-referenced generation of 10 GHz microwaves and calibration of 100 GHz. **a** Hardware configuration. **b** RF spectrum of generated 10 GHz microwave. **c** Phase noise spectra of 10 GHz microwaves generated with different conditions. **d**, **e** Calibration of 100 GHz microwaves with the slave comb in a free-running state (**d**) and after stabilization (**e**) for comparison. BPF Bandpass filter, PD Photodetector, AWG Arbitrary waveform generator, FMC Frequency multiplier chain, PCA Photo-conductive antenna, and RBW Resolution bandwidth

As for another verification, the slave comb was utilized for microwaves generation through higher-order RF harmonics of its repetition rate of 100.0 MHz. The generation scheme (Fig. 4a) consists of a high-speed photo-detector (PD) of a 25 GHz bandwidth (RXM25AF, Thorlabs) and a bandpass filter (BPF), which creates a 10 GHz signal by extracting the 100^th^ harmonic of the slave comb’s repetition rate. The 10 GHz microwave signal showed a high signal-to-noise ratio of 68 dB with a resolution bandwidth of 2 kHz (Fig. 4b) when evaluated using an RF spectrum analyzer (E4448a, Agilent) with the remote comb-to-comb stabilization turned on. Then, using an FFT spectrum analyzer (SR760, Stanford Research Systems), the residual phase noise of the 10 GHz signal was measured with high precision by taking another 10 GHz signal produced from the master comb as the reference local oscillator^34^. The result (Fig. 4c) showed a phase noise level –25 dBc Hz^–1^ in a free-running state of the slave comb, which reduced to as low as –80 dBc Hz^–1^ at 1 Hz offset frequency when the comb-to-comb stabilization was turned on. Note that the optical phase noise of the out-of-loop frequency ν_3_ was also measured between the slave and master combs and scaled down to 10 GHz for comparison to the phase noise of the slave comb (Fig. 4c). In addition, the phase noise of the master comb itself was obtained between two 10 GHz microwaves extracted at different spectral locations, one at 1531 nm and the other at 1564 nm, of the master comb. As a result, it is concluded that our remote comb-to-comb stabilization permits the slave comb to be used as a stable microwaves generator without notable phase noise increase within the shot noise limit of the used photo-detector.

Lastly, the slave comb was tested for a calibration of 0.1 THz waves, ten times higher than the previous microwaves, which was synthesized electrically through a frequency multiplication chain (FMC, SGX WR8.0 with multiplication factor = 9, Virginia Diodes). The terahertz calibration scheme (Fig. 4a) was configured to mix the 100 GHz waves under test directly with the slave comb through a photo-conductive antenna (TERA 15-RX-FC, Menlo Systems). Beat frequencies with the 1000^th^ harmonic of the slave comb were recorded using a digitizer, while the terahertz waves were modulated using an arbitrary waveform generator (AWG) through a programmed sequence of 250 frequency positions following a word pattern of ‘KAIST’ spanning a 0.45 kHz range from a bottom of 100 GHz. Test results revealed that when the slave comb was in a free-running state, the calibrated values were drifted by an amount of 260 Hz over 20 s (Fig. 4d). On the other hand, with the remote comb-to-comb stabilization turned on, the calibrated frequency positions remained stable within a digitizer-limited resolution of 3 Hz, with a two-order improvement over the same period of observation (Fig. 4e). This confirmed that the slave comb suffices to calibrate ultra-high microwaves to be used in next generations of wireless mobile communications.

## Discussion

It is concluded that our scheme of comb-to-comb stabilization was effective in transferring the frequency stability of the master comb, endowed by stabilization to a high-finesse optical cavity, over to a remote slave comb through a 1.3 km atmospheric FSO link. The transferred stability is estimated to be a 15-digit value, being verified by out-of-loop comparison with a residual instability of 1.2 × 10^–15^ at 1.0 s averaging between the master and slave combs. Note that our FSO link itself was capable of offering the frequency instability on the order of 1.7 × 10^–15^ at 0.1 s averaging, as it was calculated from the measured optical phase noise (Fig. 1e). This residual instability is considered near the limit imposed ultimately by atmospheric-turbulence-driven reciprocity breakdown. Nonetheless, with increasing the averaging time up to 100 s or more, the overall residual instability reduces below the absolute accuracy of even the best optical clocks reported today.

The remotely stabilized slave comb was tested as a wavelength ruler to calibrate a tunable laser operating at a wavelength scanning speed of 100 nm s^–1^ over a 50 nm spectral range for broadband molecular absorption spectroscopy. In addition, the slave comb was employed to generate 10 GHz microwaves with a phase noise spectral density of –80 dBc Hz^–1^ at 1 Hz offset over a –115 dBc Hz^–1^ noise floor, and to detect 0.1 THz millimeter waves with a fine resolution of 3 Hz. Convincingly, our free-space comb-to-comb stabilization is expected to expedite the atmospheric transfer of optical clock signals for diverse long-distance ground-to-ground or even ground-to-satellite applications.

## Methods

### Master comb stabilization

The master comb was built by stabilizing an Er-doped fiber oscillator to a high-finesse cavity made of very low thermal expansion material. One comb line was extracted by injection locking to a laser diode with an output power of tens of mW without loss of the original comb frequency stability. The extracted comb line was locked to a resonance dip of the high-finesse cavity by adjusting the carrier-envelope offset *f*_0_ through the Pound-Drever-Hall (PDH) control. Simultaneously, by devising an *f-2f* interferometer, the repetition rate *f*_r_ was regulated to stabilize the whole mater comb lines with a zero carrier-envelope offset. The master comb was set to act as a frequency synthesizer producing multiple frequency signals of a narrow linewidth of 1.0 Hz at 1.0 s averaging, with each output frequency signal being individually selectable with a 0.1 GHz increment over a bandwidth of 4.25 THz around a 1550 nm center wavelength. Further details are available in an authors’ separate publication of ref. ^25^.

### FSO link construction

The optical link was set up on the KAIST main campus in Daejeon, South Korea. The transceivers installed at Site A and Site B were identical, each being a bi-static transmitter-to-receiver telescope pre-aligned symmetrically to maximize the optical power transmission. In mild weather conditions, the optical power arriving at the receiving fiber was adjusted to maintain an average of 100 μW. Transmission breakdown occurred when the received optical power falls below a threshold limit of 0.5 μW, which was avoided by subsequent EDFA-based power amplification of 20 dB. The transceiver-to-transceiver power attenuation was estimated to be 26 dB, including a 6 dB power loss in light focusing onto the single-mode fiber coupler from free space. There was additional power loss of ~20 dB caused by the ground fiber networks of RF & optical signal processing.

### Slave comb stabilization

The master comb at Site A was stabilized to a repetition rate of 100.0 MHz. As for the slave comb, two distinct combs were set up at Site B; one was a nonlinear-polarization-rotation type of a repetition rate (*f*_*r*′_) of 100.0 MHz (C-fiber, Menlosystems GmbH) and the other was a saturable-absorber-assisted soliton type with a repetition rate (*f*_*r*″_) of 203.8 MHz. The latter comb had a narrow spectral bandwidth of 4.0 nm, which was extended through an EDFA and a highly nonlinear fiber (Fig. 2b) to be phase-locked to the transferred frequencies of ν_1_ (1531 nm) and ν_2_ (1564 nm). Specifically, when the repetition rate of the slave comb needs to be different from that of the master comb, the RF beat notes of *ν*_1_ & *ν*_2_ with the slave comb have to be adjusted independently until the slave comb’s repetition rate settles into a wanted value. Otherwise, the RF beat notes are locked simply to an identical offset value.

## Data Availability

The data that support the plots within this paper and other findings of this study are available from the corresponding author upon reasonable request.
